# Upper limb dimensions in adults presenting for elective surgery – implications for blood pressure measurement

**DOI:** 10.1186/s12871-020-00994-z

**Published:** 2020-04-04

**Authors:** Christopher Chow, Peter Ceglowski, Katie Lehane, Anita Pelecanos, Kellie Wren, Victoria A. Eley

**Affiliations:** 1 Faculty of Medicine, The University of Queensland, Brisbane, Queensland Australia; 2 QIMR Berghofer Medical Research Institute, Brisbane, Queensland Australia; 3 Department of Anaesthesia and Perioperative Medicine, Royal Brisbane and Women’s Hospital, Butterfield St, Herston, Queensland 4006 Australia

**Keywords:** Arm shape, Blood pressure, Measurement, Perioperative

## Abstract

**Background:**

Arm conicity is associated with non-invasive blood pressure (NIBP) measurement error and may be avoided by using finger cuffs. Predicting arm conicity may help decisions regarding NIBP measurement techniques.

**Methods:**

We obtained upper limb measurements of adults presenting to the Pre-Anesthetic Clinic to determine: the suitability of arm and finger cuff sizes; the best anthropometric predictor of arm conicity based on the right arm slant angle; the incidence of a right arm slant angle < 83 degrees. Right mid-arm circumference (MAC) was compared to recommended cuff sizes and finger circumference compared to available cuffs. Slant angle was calculated from the measurements obtained. Linear regression was used to determine the better predictor of right arm slant angle. Correlation coefficients were calculated and R^2^ values compared.

**Results:**

Four hundred fifty-four patients participated and 453 had cone-shaped arms. One participant (0.2, 95% CI 0.0–1.2) had a MAC outside the recommended cuff range. Twenty-five participants (5.5, 95% CI 3.6–8.0) had a middle finger circumference greater than the largest ClearSight™ cuff. Body mass index (BMI), weight and right MAC all had low to moderate correlation with right arm slant angle (r = − 0.49, − 0.39, − 0.48, all *p* < 0.001) and regression revealed R^2^ values of 0.24, 0.15 and 0.23. Six participants (1.3, 95% CI 0.5–2.9) had a slant angle < 83 degrees.

**Conclusion:**

Current NIBP equipment caters for most patients, based on the traditional measure of MAC. The utility of finger cuffs is limited by cuff size. BMI and right MAC showed the most promise in predicting arm conicity.

## Background

Non-invasive blood pressure (NIBP) measurement is an essential component of perioperative care [1]. Accurate pre-operative measurement allows optimal preparation for elective surgery [2]. Accurate intraoperative and post-operative measurement permits detection and diagnosis of conditions presenting with hypertension (pain, drug overdose, hypertensive crises) as well as conditions causing hypotension (hemorrhage, infection cardiac ischemia, drug overdose). Timely diagnosis and treatment of these conditions is an important responsibility of the perioperative physician and anesthetist.

Current methods of NIBP measurement rely on an arm cuff – through application of the intermittent auscultatory method [3] or more commonly in the intraoperative and post-operative environment, the intermittent automated oscillotonometric technique [4]. The influence of cuff bladder width and length on the accuracy of obtained readings is well known [5] and the American Heart Association (AHA) provides recommended bladder width and cuff sizes, based on a patient’s mid-arm circumference (MAC) [6].

**Fig. 1:**
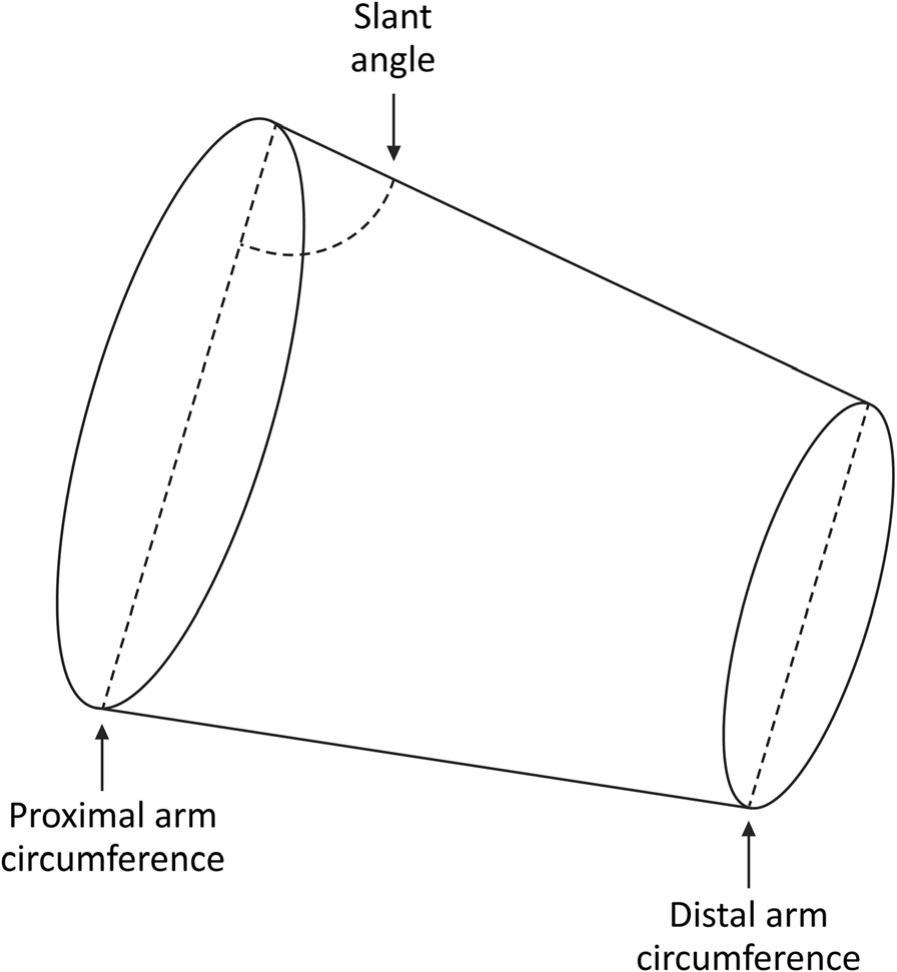
Diagram depicting the arm as a truncated cone, adapted from Palatini et al. [8] The slant angle, upper and lower arm circumference are identified

Recently the role of arm shape, or conicity, has been acknowledged as an additional factor influencing the accuracy of NIBP readings [7–9]. The degree of conicity can be expressed by calculating the slant angle [8]. If the arm is considered a truncated cone (frustum), the slant angle is the angle between the slant of the cone and the base (the circumference at the axilla). See Fig. 1. As the arm becomes more cone-shaped, the slant angle becomes smaller. Palatini et al. demonstrated that the difference between readings obtained from a conical cuff and those obtained from a standard rectangular cuff were greatest when the slant angle was less than 83 degrees [8]. Alternative methods of NIBP measurement use finger cuffs, which are not affected by the variation in size and shape of the arm. ClearSight™ and CNAP™ are two such devices, providing continuous NIBP measurement using finger-cuffs in a range of sizes.

In this exploratory study, we aimed to describe the arm size and shape of adults presenting for elective surgery at an Australian tertiary referral hospital. We aimed to describe the required cuff sizes according to the AHA recommendations [6]; to compare the finger circumference of the middle finger to ClearSight™ and CNAP™ finger cuff sizes; to evaluate easily measured anthropometric predictors of right arm conicity, based on the right slant angle; and the proportion of patients with a right arm slant angle < 83 degrees. We also aimed to evaluate the experience of participants in terms of cuff placement and skin bruising related to arm cuff use.

## Methods

This manuscript complies with STROBE reporting recommendations for observational studies. This prospective observational study was approved by the ethics committee of The Royal Brisbane and Women’s Hospital (HREC /18/QRBW/335) and written informed consent was obtained from all participants. Participants were recruited from the Pre-Anesthetic Clinic of the Royal Brisbane and Women’s Hospital, where patients undergo surgical, anesthesia, nursing and pharmacy assessments prior to elective surgery. Not all pre-operative patients attend the clinic; patient selection for attendance is based on patient co-morbidities and surgical complexity. Baseline data was collected including age, sex, self-reported ethnicity, current diagnosis of hypertension, current or previous diagnosis of ischemic heart disease, and current use of antihypertensive medications. Data on ethnicity was recorded due to the known influence of ethnicity on body habitus [10]. Details of the surgical sub-specialty team that was scheduled to perform the elective procedure was recorded, along with the participant’s body mass index (BMI) calculated from their weight in kilograms and height in meters measured on the day of recruitment.

Arm and finger measurements were obtained from both arms, by three trained operators, using a standard medical measuring tape (Prestige Fibre Glass Tape Measure™, Prestige Medical, Northridge California). Operators were trained according to the recommendations of the National Health and Nutrition Examination Survey Anthropometry Procedures Manual. When available, measurements were taken according to standard anthropometric measurements, using bony landmarks for reproducibility [11]. Two measurements of arm length were obtained. Arm length A (a standard measurement) was measured with the participant standing with the elbow flexed and held by the side, on the posterior aspect of the arm. The length was measured from the uppermost edge of the posterior border of the spine extending from the acromion process, to the olecranon process [11]. At the mid-point of arm length A, the MAC was measured. With the participant standing and the arm hanging loosely by the side, arm length B (a non-standard measurement) was obtained, measured on the medial aspect of the arm, from the axilla to the antecubital fossa [7]. This non-standard measurement was obtained in order to calculate the slant angle of the arm in the area that a NIBP cuff is usually placed. With the arm remaining by the side, the proximal arm circumference (non-standard measurement) was obtained at the axilla and the distal arm circumference (non-standard measurement) was obtained just above the elbow crease. These non-standard measurements were used in the calculation of the slant angle. The finger circumference (non-standard measurement) was measured at the mid-point of the middle phalanx of the middle finger, with the hands resting on a table.

The right MAC measurements were compared with the AHA-recommended NIBP cuff sizes, which are based on MAC [6]. The right middle finger circumference measurements were compared with the largest available ClearSight™ and CNAP™ finger cuff sizes (up to 6.8 cm and 8.8 cm circumference respectively). The section of the arm where a NIBP cuff is placed can be considered mathematically as a truncated cone (frustum) [7, 8]. See Fig. 1. Cone-shaped arms were defined as those in which the proximal arm circumference was greater than the distal arm circumference. Only those participants with cone-shaped arms were included in the calculations regarding arm slant angle. The slant angle is the angle created between the largest base of the cone (described by the circumference measured at the axilla) and the angle of the slant of the cone. The slant angle can be calculated: slant angle = arccosine[(C1- C2)/(2.π.L)] x (360/2π) in which ‘C1’ is the proximal arm circumference, ‘C2’ is the distal arm circumference, and ‘L’ is arm length B [8]. The slant angle was calculated from the obtained measurements.

Participants were asked to respond to two statements relating to their experience of having their blood pressure measured. They responded “never”, “sometimes” or “always” to the statements: “When nurses or doctors take my blood pressure they put the cuff on my lower arm or leg”; “When nurses or doctors take my blood pressure it causes bruises to my skin”. Presentation of survey questions is shown in Supplemental Figure S1.

The sample size for this exploratory study was one of convenience, aiming to be large enough to be representative of the population of patients attending the Pre-Anesthetic Clinic. Continuous participant characteristics were summarized using the range with mean (SD) or median (IQR) and categorical participant characteristics with number (percent). Independent t-tests were used to test for differences in arm measurements between males and females and paired t-tests were used to test for differences in arm measurements between left and right arms. The associations between right arm slant angle and the anthropometric measures BMI, weight and right MAC were linear and explored using Pearson correlation coefficients. Right arm slant angle was modelled using simple linear regression. Separate models were created for BMI, weight and right MAC. The R^2^ values of these three models were compared to identify the important predictors of the variance in right arm slant angle. A statistical significance threshold was set at α < 0.05. Data were analyzed in STATA Statistical Software Release 15. Responses to the survey questions were reported as a number (percent).

## Results

**Fig. 2:**
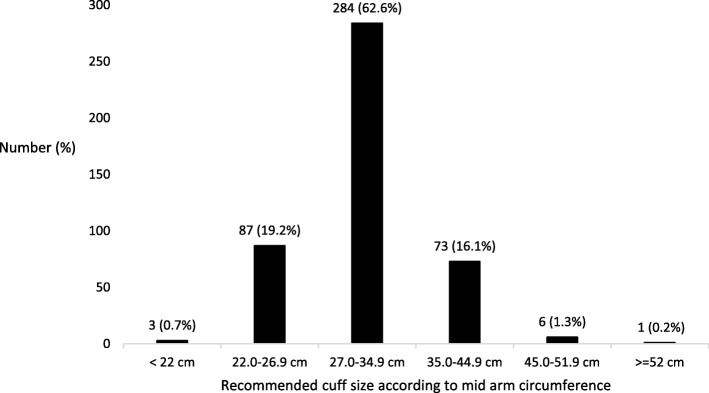
Frequency of recommended cuff sizes according to right mid-arm circumference, [6] 454 adults presenting for elective surgery

Four hundred and fifty-four participants were recruited between November 2018 and February 2019. They had a mean (SD) age of 59.9 years (16.6), 247 (54.4%) were female and 409 (92.1%) were Caucasian. They had a median (IQR, range) BMI of 28.1 kg/m^2^ (24.2–33.4, 16.1–60.9). Plastic surgery was the most common surgical sub-specialty (130, 28.6%) followed by general surgery (71, 15.6%) and urology (65, 14.3%). The remaining 19 sub-specialties each comprised less than 6% of participants. Table 1 shows the co-morbidities, antihypertensive use and arm and finger measurements of the participants. Figure 2 shows the distribution of recommended NIBP arm cuff sizes according to measured right MAC. One participant (0.2, 95% CI 0.0–1.2) had a MAC outside the recommended cuff range. Twenty-five participants (5.5, 95% CI 3.6–8.0) had a middle finger circumference > 6.8 cm and none had a middle finger circumference greater than 8.8 cm.

**Table 1:** Co-morbidities, antihypertensive use and arm and finger dimensions, 454 adults presenting to the Pre-Admission Clinic

Characteristic	Number (%)
Diagnosed ischaemic heart disease	63 (13.9)
Current diagnosis of hypertension	197 (43.4)
Antihypertensive use (*n* = 162)	
Single agent	84 (51.9)
Two agents	66 (40.7)
≥ 3 agents	12 (7.4)
Right mid-arm circumference cm, mean (SD) [range]	31.0 (5.0) [20.8–52.5]
Right slant angle degrees, mean (SD) [range]^a^	86.9 (1.3) [80.3–90.5]
Right middle finger circumference cm, mean (SD) [range]	5.8 (0.6) [3.9–7.6]

^a^*n* = 453

There was no difference between males and females regarding the right MAC (mean difference 0.05 cm, 95% CI -0.89 – 0.98, *p* = 0.92) or the right arm slant angle (mean difference − 0.13 degrees, 95% CI -0.37 – 0.12, *p* = 0.30). There was a statistically significant difference between the left and right arm MAC, with a mean difference of 0.40 cm (95% CI 0.28–0.51, *p* < 0.001). There was no difference between left and right arm slant angle, with a mean difference of − 0.02 degrees (95% CI -0.08 – 0.03, *p* = 0.37). Six participants (1.3, 95% CI 0.5–2.9) had a right arm slant angle < 83 degrees. Table 2 shows the correlations and individual regression models for the outcome of right arm slant angle with BMI, weight and right MAC. The explanatory variables each show low to moderate correlation with right arm slant angle. BMI explained 24% of the variation in right arm slant angle, similar to right MAC (23%).

**Table 2:** Relationship between right slant angle B and participant body mass index, weight and mid arm circumference, 453 adults with cone-shaped arms presenting to the Pre-Admission Clinic

Correlation			Regression models Outcome: right arm slant angle		
	r	*P*-value	Intercept	β (Slope) (95% CI)	R^2^
BMI kg/m^2^	−0.49	< 0.001	89.45	−0.09 (− 0.10 – − 0.07)	0.24
Weight kg	− 0.39	< 0.001	88.78	− 0.02 (− 0.03 – − 0.02)	0.15
Right MAC cm	−0.48	< 0.001	90.74	−0.12 (− 0.14 – − 0.10)	0.23

*BMI* body mass index

*MAC* mid arm circumference

Thirty-six (7.9, 95% CI 5.6–10.8) responded that the cuff had been placed on their lower arm or leg “sometimes” or “always” and 14 (3.1, 95% CI 1.7–5.1) responded that blood pressure measurement caused bruising to the skin “sometimes” or “always”.

## Discussion

Our results suggest that the current range of NIBP cuff size is suitable for the majority of patients presenting to the Pre-Anesthetic Clinic. Only 0.2% of participants were outside the recommended arm cuff range [6] but 5.5% had a finger circumference that would be too large for the largest ClearSight™ finger cuff. While the CNAP™ finger cuff would suit all participants, the arm cuff that is used to calibrate the CNAP™ device (designed for maximum MAC of 40 cm) would not be suitable for 24 participants (5.3%). Based on arm conicity, 1.3% of patients may be expected to have inaccurate blood pressure measurements. Of the three easily measured anthropometric predictors we evaluated, BMI and right MAC showed the most promise in helping to predict arm conicity, however these accounted for only 24 and 23% of the observed variation respectively. Less than 10% of the cohort reported cuff placement other than the arm and skin bruising.

Our population had a high rate of diagnosed hypertension of 43%, higher than reported population rates of 34% for Australia [12]. This is likely to be related to the mean age of the cohort and clinical selection criteria for attendance of patients at the clinic. However it does highlight the risk profile of patients presenting for surgery in our institution and the importance of accurate blood pressure measurement. The mean BMI of this cohort was in the overweight category according to the World Health Organization classification [13] however the BMI range of 16.1–60.9 indicates a wide range of body morphology in patients presenting for care at our institution.

To our knowledge, this is the largest evaluation of arm measurements specific to NIBP measurement equipment in a pre-operative population. Obtaining these measurements has allowed us to determine if current equipment meets the needs of patients presenting for elective surgery, based on MAC. Most of our understanding of NIBP measurement error is related to the simple circumference of the arm, rather than the shape of the arm [6, 14, 15]. Data from the US, reporting on over 5000 men and over 5000 women between 2007 and 2010 reported mean MAC of 34.2 cm in men and 31.9 cm in women [16]. In those cohorts, 45% of men and 28% of women required a cuff larger than the standard adult cuff. These proportions are much higher than in our cohort (17.6% of the entire cohort) and may represent geographic and ethnicity differences. Even based on the traditional measure of MAC, our results demonstrate that there are individuals for whom the AHA recommendations simply do not cater for.

With increasing rates of obesity in Western countries, [17] there has recently been greater consideration given to the effect of obesity on not just the size, but the shape of the arm [7, 8, 18]. Subcutaneous fat distributed around the humerus influences the transmission of the brachial arterial pulse and its detection by either auscultation or oscillotonometry [14, 19]. The influence of arm shape on blood pressure measurement was explored in 1978 [20] and in 1985 Mx et al. [21] demonstrated lower blood pressure readings when a cone-shaped cuff was used. Bonso and Palatini [7, 8] introduced the concept of measuring arm conicity using simple arm measurements to obtain the conicity index (based on arm diameter) and the slant angle (based on arm circumference). Until now, their cohorts of 142 [7] and 220 individuals [8] have been the largest samples depicting arm conicity. Here we present 450 participants, specifically presenting for perioperative care, with a mean (SD) slant angle of 86.9 degrees (1.3), consistent with that described by Bonso et al. [7] of 86.2 degrees (1.6) and Palatini [8] et al. of 86.7 degrees (1.2).

This is clearly an emerging area of research and evidence of the relationship between arm conicity and NIBP measurement error remains limited. Palatini et al. suggested that greater error was observed (between standard and conical arm cuffs) when the arm conicity was less than 83 degrees. Based on that cut-off, 1.3% of our cohort would be expected to experience inaccurate measurements. While conical cuffs have been studied, [8, 15] they are not used widely and need to be studied in more diverse groups. We have identified that BMI and right MAC have a low to moderate correlation with right arm conicity described by the slant angle. Unfortunately these simple clinical measurements are unlikely to be useful predictors of arm conicity based on the slant angle, a dimension requiring multiple measurements and a cumbersome calculation.

In this study, the suitability of the finger-cuffs of the ClearSight™ and CNAP™ devices were assessed for this specific population. This was because these devices are not affected by the size and shape of the upper arm (although the CNAP™ device uses an arm cuff for calibration). As these devices also offer advanced haemodynamic monitoring (cardiac output, systemic vascular resistance) they have largely been evaluated in critical care settings [22–24]. In this context they have been shown to have good agreement with invasive blood pressure measurements [22, 23]. However there is currently no approved validation protocol for comparing these devices with NIBP measurements [15].

Our study has limitations. While we aimed to select a representative sample, selection bias may have occurred. In addition, the characteristics of patients presenting to the clinic is already biased, based on patient characteristics and the complexity of their planned surgery. While we have collected a large amount of anthropometric data, we have not related the arm size or shape to NIBP measurements or to a gold standard such as invasive arterial monitoring.

## Conclusion

We have described the arm and finger measurements of a large population of patients presenting for surgery at an Australian tertiary institution. Current NIBP equipment caters for the majority of patients, based on the traditional measure of MAC. Based on arm conicity, a slightly greater proportion may be expected to have erroneous blood pressure measurements. The utility of devices using finger cuffs is limited by the range of finger cuff sizes and the size of the calibrating arm cuff. The implications of arm conicity on the accuracy of NIBP measurement have not been well described and this topic presents an opportunity for further research.

## Supplementary information

**Additional file 1 : Figure S1.** Survey questions answered by participants, relating to their experience of having their blood pressure measured.

## Data Availability

The dataset used and analyzed during the current study is available from the corresponding author on reasonable request.

## Abbreviations

AHA: American Heart Association
BMI: Body mass index
MAC: Mid arm circumference
NIBP: Non-invasive blood pressure
STROBE: STrengthening the Reporting of OBservational studies in Epidemiology
