# Supplementary material for: Upper limb dimensions in adults presenting for elective surgery – implications for blood pressure measurement
Source: BMC Anesthesiol. 2020 Apr 4;20:76. doi: 10.1186/s12871-020-00994-z (PMC7126166; doi:10.1186/s12871-020-00994-z)
Supplement: Supplementary file 1 — Additional file 1 : Figure S1. Survey questions answered by participants, relating to their experience of having their blood pressure measured. [file 12871_2020_994_MOESM1_ESM.pdf]

3. Participant response:

When nurses or doctors take my blood pressure (please circle):

a. they put the cuff on my lower arm or leg	**Never**	**Sometimes**	**Always**
b. it causes bruises to my skin	**Never**	**Sometimes**	**Always**
